# Butyrate Improves Neuroinflammation and Mitochondrial Impairment in Cerebral Cortex and Synaptic Fraction in an Animal Model of Diet-Induced Obesity

**DOI:** 10.3390/antiox12010004

**Published:** 2022-12-20

**Authors:** Gina Cavaliere, Angela Catapano, Giovanna Trinchese, Fabiano Cimmino, Eduardo Penna, Amelia Pizzella, Claudia Cristiano, Adriano Lama, Marianna Crispino, Maria Pina Mollica

**Affiliations:** 1 Department of Pharmaceutical Sciences, University of Perugia, 06126 Perugia, Italy; 2 Centro Servizi Metrologici e Tecnologici Avanzati (CeSMA), Complesso Universitario di Monte Sant’Angelo, Via Cinthia 21, 80126 Naples, Italy; 3 Department of Biology, University of Naples Federico II, 80126 Naples, Italy; 4 Department of Pharmacy, University of Naples Federico II, 80131 Naples, Italy; 5 Task Force on Microbiome Studies, University of Naples Federico II, 80138 Naples, Italy

**Keywords:** oxidative stress, neuroinflammation, mitochondrial function, butyrate

## Abstract

Neurodegenerative diseases (NDDs) are characterized by cognitive impairment and behavioural abnormalities. The incidence of NDDs in recent years has increased globally and the pathological mechanism is not fully understood. To date, plentiful evidence has showed that metabolic alterations associated with obesity and related issues such as neuroinflammation, oxidative stress and mitochondrial dysfunction may represent an important risk factor, linking obesity and NDDs. Numerous studies have indicated a correlation between diet and brain activities. In this context, a key role is played by mitochondria located in the synaptic fraction; indeed, it has been shown that high-fat diets cause their dysfunction, affecting synaptic plasticity. In this scenario, the use of natural molecules that improve brain mitochondrial function represents an important therapeutic approach to treat NDDs. Recently, it was demonstrated that butyrate, a short-chain fatty acid is capable of counteracting obesity in an animal model, modulating mitochondrial function. The aim of this study has been to evaluate the effects of butyrate on neuroinflammatory state, oxidative stress and mitochondrial dysfunction in the brain cortex and in the synaptic fraction of a mouse model of diet-induced obesity. Our data have shown that butyrate partially reverts neuroinflammation and oxidative stress in the brain cortex and synaptic area, improving mitochondrial function and efficiency.

## 1. Introduction

In recent years, the prevalence of obesity has grown alarmingly on a global scale [1]. This condition is due to an incorrect lifestyle and to chronic high-fat diet (HFD) intake. It is well known that HFD-induced obesity often leads to low-grade chronic inflammation with a wide range of metabolic diseases [2,3,4]. The resulting metabolic inflammation has an impact on the whole body, including the central nervous system [5,6,7]. Indeed, over the last few years, a growing emphasis has been placed on the relationship between nutrition and cognitive performance. In particular, HFD appears to be linked to cognitive deficits, representing a risk factor for neurodegenerative diseases (NDDs) development [8,9].

NDDs are characterized by high levels of oxidative stress markers and by low levels of antioxidant defence in the brain. Indeed, dysregulation in the oxidant/antioxidant balance is known to be an important factor in the pathogenesis of neural dysfunctions. Cerebral cellular damage found in obesity, due to inflammation and oxidative stress, are at the basis of the development of cognitive decline processes [10,11,12]. It is well known that brain-derived neurotrophic factor (BDNF) is a neurotrophin that plays a key role in brain physiology and cognitive decline [13]. In particular, several studies show the link between HFD-induced neuroinflammatory processes and BDNF-related pathway alterations in various brain areas [14,15,16].

Cognitive decline is characterized by an alteration of neuronal connectivity and atypical synaptic plasticity [17]. The molecular mechanisms of synaptic plasticity, implying continuous remodelling of the pre- and postsynaptic areas, are crucial for the physiology and pathology of the nervous system. In this context, mitochondria, a major source of cell energy and reactive oxygen species (ROS), play an important role. In particular, the mitochondria located at synapses provide energy to support synaptic function and plasticity. Recent evidence has shown that impairment of mitochondrial function in both the brain cortex and the synaptosomal fraction is correlated with an increase in inflammatory parameters and oxidative stress leading to altered synaptic plasticity in a mouse model of diet-induced obesity [16,18]. Therefore, molecules able to modulate mitochondrial function and efficiency could represent a potential innovative approach in the prevention/treatment of neuroinflammatory processes.

Several studies have demonstrated that butyrate, a short-chain fatty acid produced by the fermentation of dietary fibre, has various beneficial effects in energy metabolism, intestinal homeostasis and immune response regulation, and it is capable of regulating different metabolic pathways at the same time [19]. Current studies have found that butyrate can relieve inflammation and enhance synaptic plasticity by reducing neuroinflammation in an animal model of Alzheimer’s disease (AD) [20] and in mouse models of Parkinson’s disease [21,22]. Several studies have emphasized that the mitochondria may be the potential target of butyrate action [23,24,25], although the underlying mechanisms are still unclear. We have recently demonstrated the ability of butyrate to decrease insulin resistance and fat accumulation in the liver of diet-induced obese mice, improving hepatic mitochondrial function and efficiency [23]. A recent study in a mouse model of epilepsy demonstrated the ability of butyrate to sustain mitochondrial function at the hippocampal level, to reduce oxidative stress and to inhibit seizures, limiting neuronal degeneration [26].

Based on this evidence, here we evaluated the effects of butyrate on neuroinflammatory state, oxidative stress and mitochondrial functions in the brain cortex and synaptic fraction of diet-induced obese mice.

## 2. Materials and Methods

### 2.1. Materials

Analytical-grade chemicals used were sourced from Sigma (St. Louis, MO, USA).

### 2.2. Animal Diet

In this experimental procedure, we used male C57Bl/6J mice (Charles River Laboratories, Calco, Lecco, Italy) housed in a temperature-standardized chamber and subjected to a daily cycle of 12 h of light and 12 h of darkness with free access to water and food. Young mice with a mean weight of 27 ± 0.8 g were used in these experiments. After a week of housing, a group (n = 6) was sacrificed early in the study for reference measurements. At the onset of the study, mice were fed the control diet (CD) or 45% high-fat diet (HFD). After 12 weeks, a subgroup of HFD mice (n = 6) was treated by gavage with butyrate (100 mg/kg q.d.) for 6 weeks, while the remaining CD and HFD mice received water as vehicle. The dose of butyrate was selected from a previous study, using the same experimental conditions [23]. At the conclusion of the treatment period, the animals were anaesthetised with chloral hydrate (0.040 g/100 g bw) and sacrificed by beheading. Blood samples were taken from the inferior cava, centrifuged at 1000× *g* for 10 min and stored at −80 °C for subsequent testing. The extracted brain cortex that was not used to prepare synaptosomes and mitochondria was frozen and stored at −80 °C for later analysis.

### 2.3. Body Composition and Energy Balance

During treatments, body weight and food consumption were checked daily to calculate body weight gain and gross energy assumption. The energy balance assessment was carried out during the 18 weeks of diet treatment by comparative carcass evaluation [23].

Bomb calorimetry was used for the determination of the gross energy density of CD and HFD (15.8 and 21.9 KJ/g, respectively) and of the energy density of faeces and the carcasses (Parr adiabatic calorimetric; Parr Instrument Company, Moline, IL, USA). Metabolizable energy (ME) intake was calculated by subtracting the energy measured in faeces and urine from the gross energy intake. The gross energy assumption was assessed using daily dietary intake and gross energy density. The assessment of energy content, fat, protein and water percentage in animal carcasses was carried out according to a published protocol [23]. In brief, aliquots of the carcass were homogenated and used for the determination of these parameters. The water content was derived from the difference between the weight of the homogenate before and after drying at 70 °C in a vacuum oven. Lipid content was obtained gravimetrically by chloroform/methanol extraction and constant weight evaporation with a rotating evaporator (Heidolph, Kelheim, Germany) by the method described by Folch et al., 1957 [27]. Lipid energy was determined from the content of lipids, using the coefficient of 39.2 kJ/g. The content of protein was measured as described by Brooks et al., 1995 [28] and converted to energy as protein by using the value of 23.5 kJ/g.

Energy efficiency was determined as the percentage of body energy gain per ME intake, while the energy expenditure was calculated subtracting body energy gain from ME intake. The energy gain of the body was determined as the difference between the body’s energetic content at the end of the experimental period and the energy content of the mice killed at the start of the experimental period (baseline measurements).

### 2.4. Serum Parameters

Serum triglyceride and cholesterol values were measured using colorimetric enzyme methods with commercial kits (SGM Italia, Rome, Italy, and Randox Laboratories Limited, Crumlin, UK). Commercially available ELISA kits were used to determine the serum concentrations of interleukin (IL)-1β, tumour necrosis factor-α (TNF-α) (BioVendor, Brno, Czechia), adiponectin and leptin (B-Bridge International, Mountain View, CA, USA).

### 2.5. Brain and Synaptosomes Parameters

In order to evaluate the peroxidation of lipids in the cerebral cortex homogenate and in the synaptosomal fraction, we measured the malondialdehyde (MDA) concentration by the thiobarbituric acid method according to a published protocol [29]. In brief, synaptosomes and cerebral cortex tissue were homogenized in 1.15% KCl solution. An aliquot of the homogenate was added to a reaction mixture containing SDS 8.1%, acetic acid 20% (pH 3.5), thiobarbituric acid 0.8% and distilled water. Samples were then boiled for 1 h at 95 °C and centrifuged at 3000× *g* for 10 min. The absorbance of the supernatant was assessed by spectrophotometry at 550 nm and MDA concentration was calculated. The levels of ROS were determined as previously reported [30]. Aliquots of synaptosomes and brain tissue homogenate were diluted in 100 mM potassium phosphate buffer (pH 7.4) and incubated with dichlorofluorescein diacetate (Sigma-Aldrich, St. Louis, MO, USA) in dimethyl sulfoxide for 15 min at 37 °C, obtaining a final concentration of 5 µM. Subsequently, the samples were centrifuged at 12,500× *g* for 10 min at 4 °C. The pellet was mixed at ice-cold temperatures in 5 mL of 100 mM potassium phosphate buffer (pH 7.4) and incubated for one hour at a temperature of 37 °C. The fluorescence was measured at 488 nm and at 525 nm wavelengths for excitation and for emission, respectively. ROS were quantified by dichlorofluorescein standard curve in dimethyl sulfoxide (0–1 mM). The levels of TNF-α and IL-1β, IL-6 and IL-10 in the cerebral cortex homogenate and in the synaptosomal fraction were determined by ELISA analysis as previously reported [3]. The dithionitrobenzoic acid-GSSG reductase recycling assay was used to assess the levels of reduced glutathione (GSH) and oxidized glutathione (GSSG) in the homogenates of cerebral cortex and in synaptosomal fractions. The ratio between GSH and GSSG was used as marker of oxidative stress [31].

### 2.6. Preparation of Mitochondria and Synaptosomes from the Cerebral Cortex

Synaptosomes were prepared according to the standard procedure [32] which yields a well-characterised synaptosomal fraction [33,34,35,36]. This fraction was routinely tested to verify that the synaptosomal expression levels of presynaptic proteins were significantly enriched compared to the corresponding homogenate. In summary, the cerebral cortex was rapidly dissected and homogenized in isotonic medium (HM) containing 0.32 M sucrose and 10 mM Tris-Cl, pH 7.4. Subsequently, the homogenate was centrifuged at 2000× *g* for 1 min at 4 °C and the sediment was resuspended in HM and centrifuged under identical conditions to produce a sediment that contains nuclei, cellular debris and large synaptosomes (P1). The supernatant fraction was removed and centrifuged at 23,000× *g* for 4 min at 4 °C to produce a sediment that was in turn resuspended in HM and centrifuged again at 23,000× *g* for 4 min at 4 °C to obtain a second sediment containing synaptosomes, free mitochondria and myelin fragments (P2).

Differential centrifugation of the P2 aliquots was necessary for the preparation of isolated synaptosomes and mitochondria. The synaptosomal fraction was obtained by fractionating the P2 by discontinuous gradient of Ficoll. The P2 fraction, concentrated at 3.5 mg/mL, was distributed on a 5% and 13% discontinuous Ficoll gradient, dissolved in HM and centrifuged at 45,000× *g* for 45 min at 4 °C. The purified synaptosomal fraction was collected at the interface of the two Ficoll layers, diluted with nine volumes of HM and sedimented by centrifugation (23,000× *g* for 20 min at 4 °C). The sediment was homogenized into HM and the concentration of protein was determined using Bradford colorimetric assay with bovine serum albumin (BSA) as standard. To isolate mitochondria, the P2 fraction was centrifuged at 500× *g* for 10 min at 4 °C in a medium consisting of 50 mM HEPES, 80 mM LiCl, 1 mM EGTA, 5 mM Tris-PO4 and 0.1% (*w*/*v*) fatty-acid-free BSA, pH 7.0. The supernatant was centrifuged (10,000× *g* for 10 min at 4 °C) and the pellet was resuspended in a medium consisting of 50 mM HEPES, 80 mM LiCl, 1 mM EGTA, 5 mM Tris-PO_4_ and 0.1% (*w*/*v*) fatty-acid-free BSA, pH 7.0.

The protein content of isolated mitochondria was measured through Bradford colorimetric assay with BSA as standard. The quality of isolated mitochondria was guaranteed by verifying that contamination of the mitochondria by other ATPase-containing membranes was under 10% and that addition of cytochrome c (3 nmol/mg protein) enhanced only state 3 respiration by approximately 10%, in accordance with previous reports [4].

### 2.7. Measurements of Mitochondrial Oxidative Capacities and Degree of Coupling

Oxygen used by isolated mitochondria was determined using the Hansatech high-resolution respirometry oxigraph (Yellow Spring Instruments, Yellow Springs, OH, USA) according to a published protocol [16]. Briefly, the oxygen consumption from isolated mitochondria were assessed in the presence of 10 mM succinate plus 3.75 mM rotenone or 10 mM pyruvate plus 2.5 mM malate in a medium (pH 7.0) consisting of 80 mM KCl, 5 mM KH_2_PO_4_, 50 mm HEPES, 1 mm EGTA and 0.1% (*w*/*v*) fatty-acid-free BSA. State 4 oxygen consumption was detected without ADP, while state 3 oxygen consumption was evaluated in the presence of 0.3 mM ADP. The respiratory control ratio (RCR) was obtained as the ratio between states of respiration 3 and 4 as previously reported [37]. The coupling degree was calculated in the cerebral mitochondria incubated in presence of succinate (10 mM) and rotenone (3.75 mM), and applying the equation of Cairns et al. [38],

degree of coupling = √ 1− (Jo)_sh_/(Jo)_unc_

(Jo)_sh_ is the rate of oxygen consumption (OCR) obtained after the addition of oligomycin (2 mg/mL), an inhibitor of ATP synthase; (Jo)_unc_ is the maximal OCR obtained in the presence of the uncoupler carbonyl cyanide-p-trifluoromethoxyphenylhydrazone (1 mM, FCCP), which undoes the trans-mitochondrial proton gradient. The enzymatic activity of aconitase and superoxide dismutase (SOD) was measured spectrophotometrically as previously reported [4,39].

### 2.8. Seahorse XFp Analyzer Cell Mito Stress Test

The measurements of OCRs in the synaptosomal fraction of the mice cortex were carried out with the Seahorse XFp Analyzer (Seahorse Biosciences, North Billerica, MA, USA) using Cell Mito Stress Test kit (Seahorse Bioscience, 101706-100) according to a previously published protocol [16]. In summary, poly-D-lysine was used to coat the XFp plates. Synaptosomal protein (10 mg) were seeded in each well in a final volume of 100 µL of ionic medium (20 mM HEPES, 10 mMD-Glucose, 1.2 mM Na_2_HPO_4_, 1 mM MgCl_2_, 5 mM NaHCO_3_, 5 mM KCl, 140 mM NaCl, pH 7.4 at 4 °C). The plates were centrifuged (2000× *g* for 1 h at 4 °C) and the medium was changed with 180 µL of Seahorse incubation medium (3.5 mM KCl, 120 mM NaCl, 1.3 mM CaCl_2_, 0.4 mM KH_2_PO_4_, 1.2 mM Na_2_SO_4_, 2 mM MgSO_4_, 4 mg/mL BSA, 15 mM D-glucose, 5 mM pyruvate, 2.5 mM malate, pH 7.4 at 37 °C).

Basal respiration was evaluated in the presence of the incubation medium alone. The proton leak was assessed after the inhibition of ATPase by oligomycin. ATP production was derived from decreased respiration by inhibiting ATP synthase with oligomycin. Afterward, maximal mitochondrial respiration was measured by the addition of FCCP. Finally, extra-mitochondrial respiration was measured after the addition of antimycin A and rotenone, inhibitors of the complexes III and I, respectively. Coupling efficiency is the oxygen consumed for the synthesis of ATP compared to that driving proton leak and was measured as the fraction of basal mitochondrial OCR used for ATP synthesis (ATP-linked OCR/basal OCR). Spare respiratory capacity is the ability of the cell to meet a demand for energy and was determined as the difference between the maximal and basal respiration.

### 2.9. Western Blot Analysis

The cerebral cortex and synaptosomal fraction, obtained from the various experimental groups, were lysed on ice in lysis buffer (20 mM MOPS pH 7.4, 2 mM EGTA pH 8, 5 mM EDTA pH 8, 30 mM NaF, 60 mM b-Glycerophosphate, 1 mM Sodium orthovanadate, 1% Triton X-100, 1 mM DTT) in the presence of protease inhibitors (Sigma-Aldrich). Western blot analyses were performed as previously described [40]. Briefly, proteins (20 mg/lane) were separated on 12% SDS-PAGE and electro-transferred onto a nitrocellulose membrane. Membranes were blocked at room temperature in milk buffer (1X PBS, 10% *w*/*v* non-fat dry milk, 0.1% *v*/*v* Tween-20) and then incubated at 4 °C overnight with anti-BDNF monoclonal antibody (Abcam, 1:1000) and anti-GAPDH monoclonal antibody (Merck; 1:3,000,000). Subsequently, the membranes were incubated with secondary antibody against rabbit or mouse IgG (Sigma-Aldrich; 1:20,000) for 60 min at room temperature. The signals were visualized by using the ECL system (Elabscience, Houston, TX, USA). The same membrane was used to test GAPDH expression level, used for the normalization of the data.

### 2.10. Statistical Analysis

The data are reported as means ± SEM unless otherwise indicated. The differences between the groups were compared by one-way ANOVA followed by the Newman-Keuls post-hoc test for multiple comparisons. The differences were considered as statistically significant at *p* < 0.05. Analyses were carried out with GraphPad Prism (GraphPad Software, v5.0, San Diego, CA, USA). Different letters (a,b,c) on top of the histograms indicate statistically significant differences (*p* < 0.05) among groups.

## 3. Results

### 3.1. Effect of Butyrate on Body Composition and Energy Balance of HFD Mice

**Figure 1:**
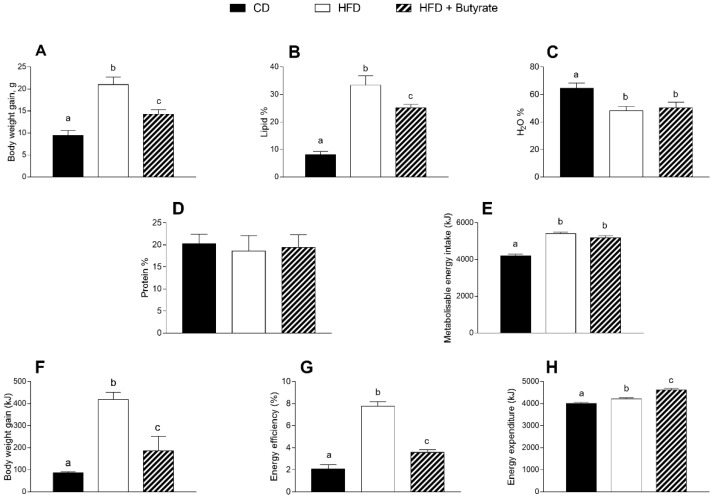
Effects of butyrate treatment on body composition and energy balance in diet-induced obese mice: (**A**) body weight; (**B**) lipid percentage; (**C**) water percentage; (**D**) protein percentage; (**E**) metabolizable energy intake; (**F**) body weight gain; (**G**) energy efficiency (%) and (**H**) energy expenditure are reported. Data are shown as means ± SEM from n = 6 animals/group. Different letters (a,b,c) on top of the bars indicate statistically significant differences (*p* < 0.05) among groups.

HFD mice were characterised by a significant increase in body weight and lipid percentage compared to control diet (CD) animals (Figure 1A,B). In addition, HFD mice exhibited a significantly lower percentage of water than CD mice (Figure 1C). Butyrate treatment, starting at 12 weeks, significantly reduced body weight and lipid content in HFD-treated mice, but did not affect the percentage of water. No variation was observed in body protein content among the three groups of mice (Figure 1D). HFD-fed groups showed a metabolizable energy (ME) intake higher than CD and butyrate had no effect on this parameter (Figure 1E). On the other hand, after butyrate treatment, a significant reduction of body weight gain and energy efficiency compared to the HFD group was observed (Figure 1F,G). Furthermore, butyrate administration caused a significant increase in the energy expenditure (Figure 1H).

### 3.2. Effect of Butyrate on Serum Metabolic Parameters and Inflammatory Markers of HFD Mice

**Figure 2:**
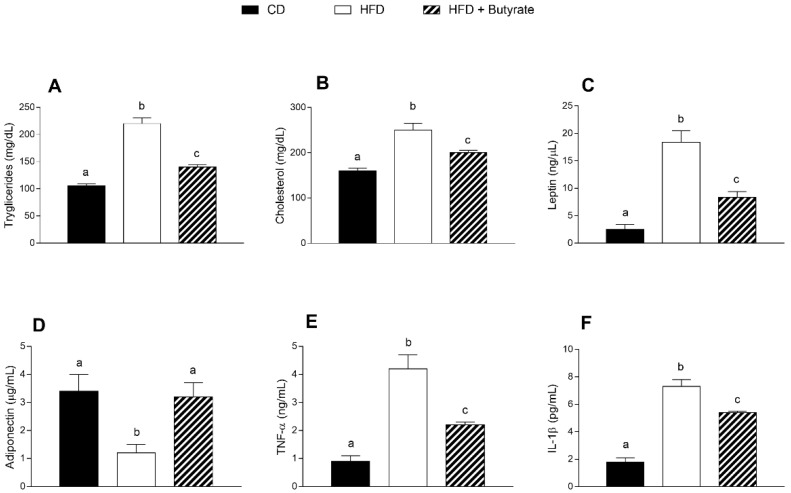
Effects of butyrate treatment on serum parameters in diet-induced obese mice: (**A**) triglycerides; (**B**) cholesterol; (**C**) leptin; (**D**) adiponectin; (**E**) TNF-α and (**F**) IL-1β serum levels are reported. Data are shown as means ± SEM from n = 6 animals/group. Different letters (a,b,c) on top of the bars indicate statistically significant differences (*p* < 0.05) among groups.

HFD mice exhibited a significant increase in triglycerides and cholesterol serum levels compared to CD animals (Figure 2A,B). The administration of butyrate significantly reduced the triglycerides and cholesterol levels in HFD-treated mice, displaying a lipid-lowering effect (Figure 2A,B). Leptin concentration was significantly increased and adiponectin content was significantly reduced in the HFD group compared to control (Figure 2C,D). The butyrate treatment decreased leptin levels and restored adiponectin levels in HFD-treated mice. The levels of pro-inflammatory cytokines, such as tumour necrosis factor-α (TNF-α) and interleukin (IL)-1β, augmented in the HFD group compared to controls (Figure 2E,F), and the administration of butyrate was able to reduce these parameters, demonstrating an anti-inflammatory effect.

### 3.3. Effect of Butyrate on Inflammation and Oxidative Stress in the Brain Cortex of HFD Mice

**Figure 3:**
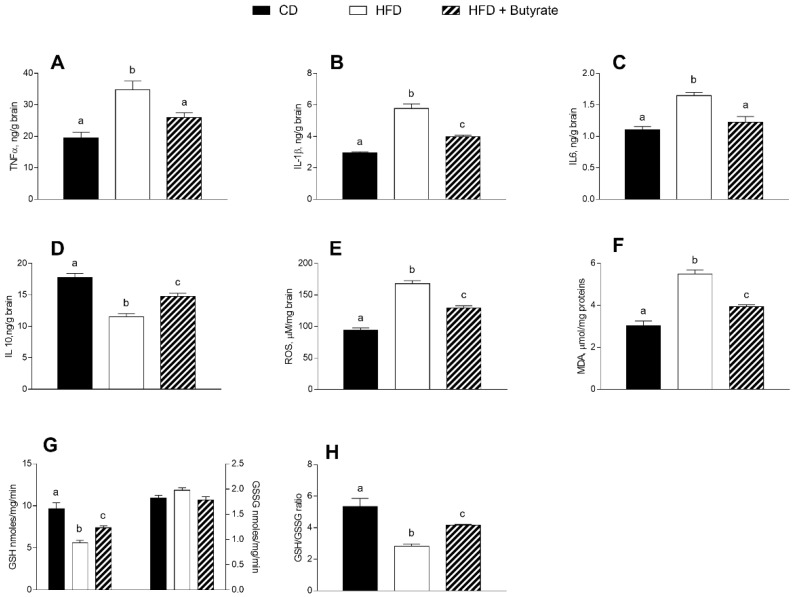
Effects of butyrate treatment on inflammation and oxidative stress in the brain cortex of diet-induced obese mice: (**A**) TNF-α; (**B**) IL-1β; (**C**) IL-6; (**D**) IL-10; (**E**) ROS; (**F**) MDA; (**G**) glutathione (GSH) and oxidized glutathione (GSSG) and (**H**) GSH/GSSG ratio are reported. Data are shown as means ± SEM from n = 6 animals/group. Different letters (a,b,c) on top of the bars indicate statistically significant differences (*p* < 0.05) among groups.

HFD induces inflammation and oxidative stress in the mouse cerebral cortex. Indeed, pro-inflammatory markers, such as TNF-α, IL-1β and IL-6, were significantly higher in the brain cortex of HFD mice compared to control animals (Figure 3A–C), while the levels of IL-10, an anti-inflammatory marker, were reduced (Figure 3D). The administration of butyrate exerted a modulatory effect on the inflammatory state. In fact, a reduction in TNF-α, IL-1β and IL-6 levels, as well as an increase in IL-10 levels were observed in the brain cortex of butyrate-treated HFD mice. In addition, in the brain cortex of the HFD group, the concentrations of ROS and malondialdehyde (MDA) were significantly higher than in the control group (Figure 3E,F), while the reduced glutathione (GSH) content and GSH-to-GSSG ratio were significantly reduced (Figure 3G,H). Butyrate improves the oxidative stress levels in HFD mice. Indeed, we observed a reduction in ROS and MDA levels and an increase in GSH content and GSH-to-GSSG ratio in butyrate-treated HFD mice. No significant differences were observed in oxidized glutathione (GSSG) content among groups.

### 3.4. Effect of Butyrate on Inflammation and Oxidative Stress in the Synaptic Fraction of HFD Mice

**Figure 4:**
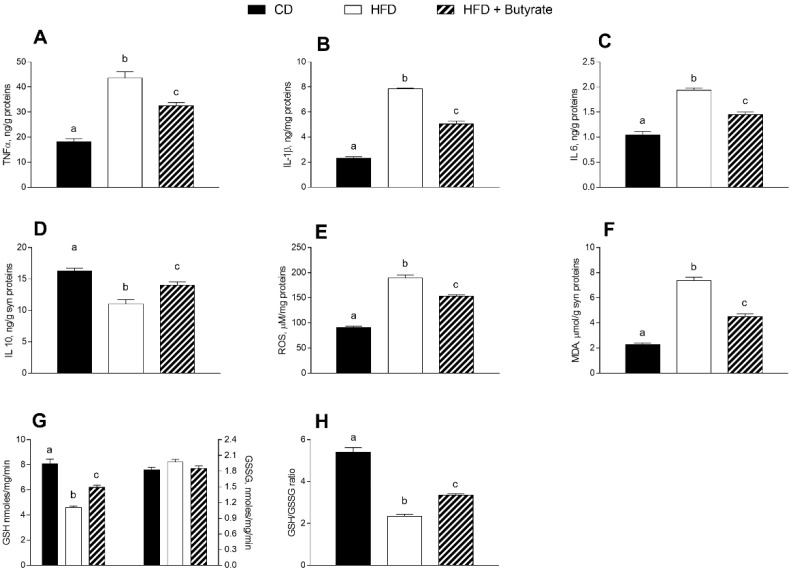
Effects of butyrate treatment on inflammation and oxidative stress in the synaptic fraction in diet-induced obese mice: (**A**) TNF-α; (**B**) IL-1β; (**C**) IL-6; (**D**) IL-10; (**E**) ROS; (**F**) MDA; (**G**) glutathione (GSH) and oxidized glutathione (GSSG) and (**H**) GSH/GSSG ratio are reported. Data are shown as means ± SEM from n = 6 animals/group. Different letters (a,b,c) on top of the bars indicate statistically significant differences (*p* < 0.05) among groups.

In the synaptosomal fraction from HFD mice brain, a higher level of pro-inflammatory markers, such as TNF-α, IL-1β and IL-6 was observed (Figure 4A–C) compared to controls, as well as a reduction in anti-inflammatory marker IL-10 (Figure 4D), indicating that the HFD induces an inflammatory state. The administration of butyrate has a modulatory effect on the inflammatory state of synaptosomes. In fact, levels of TNF-α, IL-1β and IL-6 decreased, and levels of IL-10 increased compared to the HFD group. In synaptosomes of the HFD group, an increased oxidative stress was also observed, indicated by higher concentrations of ROS and MDA (Figure 4E,F), as well as lower GSH content and GSH-to-GSSG ratio compared to controls (Figure 4G,H). When HFD mice were treated with butyrate, a decrease in ROS and MDA levels, and an increase in GSH content and GSH-to-GSSG ratio were observed. These data indicate that butyrate can improve the oxidative stress induced by a HFD in the synaptosomal fraction of mouse brain cortex.

### 3.5. Effect of Butyrate on Mitochondrial Function, Efficiency and Oxidative Stress in HFD Mouse Brain Cortex

**Figure 5:**
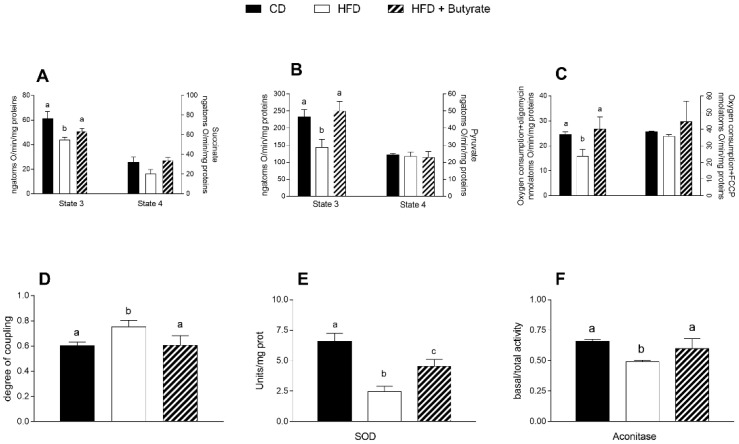
Effects of butyrate treatment on respiratory parameters of isolated mitochondria in the cerebral cortex in diet-induced obese mice: (**A**) Mitochondrial respiration rates measured in the presence of succinate and rotenone or (**B**) pyruvate and malate as substrates; (**C**) oxygen consumption in the presence of oligomycin or uncoupled by carbonyl cyanide 4-(trifluoromethoxy) phenylhydrazone (FCCP); (**D**) degree of coupling; (**E**) superoxide dismutase activities (SOD) and (**F**) aconitase activities are reported. Data are shown as means ± SEM from n = 6 animals/group. Different letters (a,b,c) on top of the bars indicate statistically significant differences (*p* < 0.05) among groups.

Mitochondria state 3 respiration, assessed using succinate or pyruvate as substrates, was significantly decreased in the brain cortex of HFD group compared to controls (Figure 5A,B). Butyrate significantly increases state 3 of respiration, in the presence of both succinate and pyruvate, compared to HFD mice. No variation was observed in state 4 respiration among all groups, using succinate or pyruvate as substrates (Figure 5A,B). To test mitochondrial efficiency, we measured oxygen consumption in the presence of oligomycin and carbonyl cyanide-p-trifluoromethoxyphenylhydrazone (FCCP) (Figure 5C). Oligomycin state 4 respiration showed a significant reduction in the brain cortex of HFD animals compared to controls, while it was significantly increased in butyrate-treated mice. No variation was found in FCCP-stimulated respiration. The energetic efficiency, assessed as the degree of coupling, was increased in HFD and decreased by butyrate treatment (Figure 5D). Activities related to superoxide dismutase (SOD) and aconitase were significantly lower in the HFD mice than in the control group (Figure 5E,F), and butyrate was able to increase these values, demonstrating its ability to promote the antioxidant defence.

### 3.6. Effect of Butyrate on Mitochondrial Function in the Synaptic Fraction from HFD Mouse Brain Cortex

**Figure 6:**
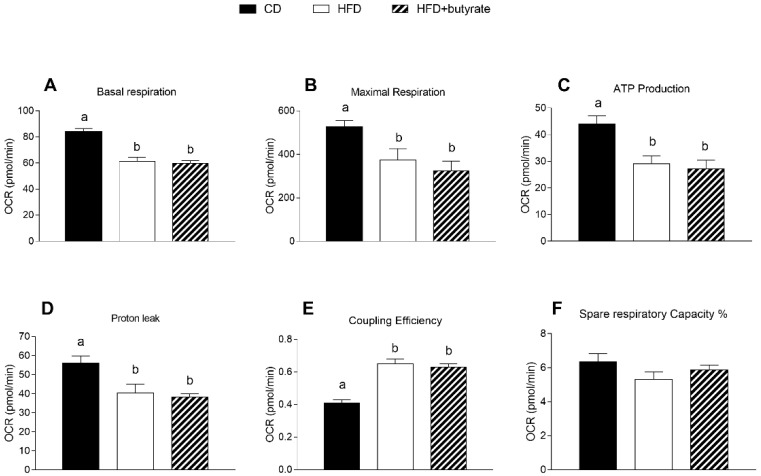
Effects of butyrate treatment on mitochondrial respiration parameters in the synaptosomal fraction in diet-induced obese mice: (**A**) basal respiration; (**B**) maximal respiration; (**C**) ATP production; (**D**) proton leak; (**E**) coupling efficiency and (**F**) spare respiratory capacity % are reported. Data are shown as means ± SEM from n = 6 animals/group. Different letters (a,b,c) on top of the bars indicate statistically significant differences (*p* < 0.05) among groups.

Results of the Cell Mito Stress Test on synaptosomal fractions showed reduced basal respiration in the brain cortex synaptosomes of HFD mice compared to controls (Figure 6A). These results were consistent with a decrease in maximal rate of respiration and ATP production in synaptosomes of HFD animals compared to the control group (Figure 6B,C). Proton leak decreased significantly and, as a consequence, coupling efficiency increased in the synaptosomes of HFD group relative to controls (Figure 6D,E). Spare respiratory capacity, i.e., the ability of the cells to respond to an energetic demand by generating ATP through oxidative phosphorylation (OXPHOS), was not different between the three groups (Figure 6F). No significant differences were observed in these respiratory parameters between HFD mice and butyrate-treated mice.

### 3.7. Effect of Butyrate on BDNF Pathways in the Brain Cortex from HFD Mouse

**Figure 7:**
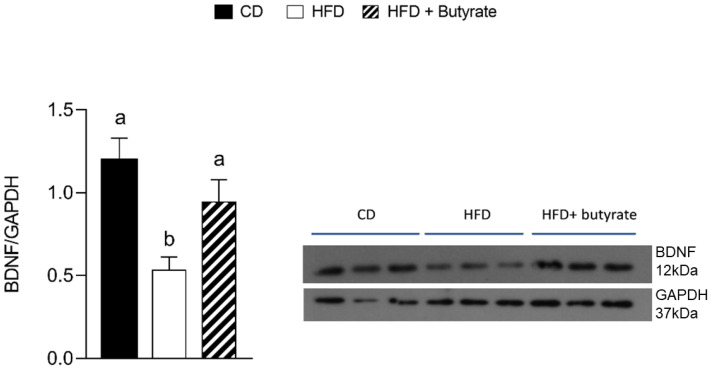
Effects of butyrate treatment on BDNF expression levels in the cerebral cortex of diet-induced obese mice. Expression level of BDNF was normalized with that of GADPH. Data are presented as means ± SEM from n = 3 animals/group. Different letters (a,b) on top of the bars indicate statistically significant differences (*p* < 0.05) among groups.

To determine the mechanism underlying the modulatory effect of butyrate on the cerebral cortex area, BDNF protein expression was evaluated. The results clearly showed that BDNF expression levels decreased considerably compared to the control group, while the administration of butyrate significantly increased BDNF expression levels (Figure 7).

## 4. Discussion

This study mainly demonstrated that the administration of butyrate in HFD-treated mice influenced cerebral bioenergetics, improving impaired brain mitochondrial functions. HFD treatment in mice led to an increase in metabolic efficiency, body weight, lipid content, metabolic alterations such as dyslipidaemia associated with increased low-grade inflammation, and a marked impairment in brain mitochondrial function. Indeed, in line with our previous results [16], we confirmed increased levels of inflammatory and oxidative stress parameters in the cerebral cortex and synaptosomal fraction of HFD mice.

Butyrate produced mainly by bacterial fermentation of fibre in the colon has been extensively investigated, from a pharmacological point of view, for its several bio-functional activities [19] and its therapeutic activities against colon cancer, ischemic stroke, hemoglobinopathies and cerebral diseases [19,41,42]. Our results showed that the administration of butyrate to HFD-treated mice results in a significant improvement of obesity-related alterations of body composition and metabolic parameters. In particular, in agreement with our previous results [23], we observed a reduction in body weight gain and fat accumulation, an improvement in metabolic alterations and a reduction in inflammatory state and oxidative stress in HFD mice. The effects of butyrate on body weight and lipids of HFD-treated mice may be due, at least in part, to increased energy expenditure and reduced metabolic efficiency. Also, the administration of butyrate improves the alterations in lipid profile and dysregulation of leptin and adiponectin levels induced by the HFD. In particular, it is well known that adiponectin is involved in lipid metabolism [43,44] and inversely correlated to inflammatory parameters and oxidative stress [45]. Indeed, following the administration of butyrate in HFD mice, we found an increase in adiponectin levels and an amelioration in pro-inflammatory cytokines serum levels. Accordingly, the ability of butyrate to downregulate cytokines and inflammatory mediators was shown [46].

A good amount of evidence suggests that inflammation, high levels of ROS and low levels of antioxidant defences in the brain can be considered potential risk factors for NDDs [47]. Butyrate has been demonstrated to have neuroprotective effects against brain injury and TNF-α-induced SH-SY5Y (Human Neuroblastoma Cell Line) neuronal cell death [48,49]. Moreover, butyrate can attenuate pro-inflammatory cytokine expression in microglia of aged mice by epigenetic gene regulation [50]. Thus, butyrate has been considered as a potential therapeutic agent against NDDs, although the molecular mechanisms through which it exerts these beneficial effects is not completely known.

In this work, we interestingly observed that the administration of butyrate was able to counteract HFD-related oxidative stress and inflammation in mice brain cortex and synaptosomes. Indeed, following butyrate administration, we observed a decrease in pro-inflammatory cytokine levels, an increase in SOD activity (the first line of defence from oxidative stress [29]) and an improvement in redox status (GSH/GSSG) in both the brain cortex and the synaptosomal fraction of HFD mice.

In selected brain regions of an AD mice model, an increased inflammatory state and oxidative stress has been demonstrated [51], indicating that obesity and AD share common metabolic alterations. Interestingly, butyrate was showed to relieve neuroinflammation and enhance synaptic plasticity in 5XFAD mice, a transgenic mouse model of AD, suggesting a protective role of butyrate in this pathology [20]. AD, as numerous neurodegenerative diseases, is characterized by synaptic area damage and related functional failure [52]. Thus, it is relevant to investigate the role of butyrate in reversing the synaptic alterations in the brain of HFD animals. Towards this aim, we used synaptosomes, subcellular vesicles that reproduce in vitro the synaptic terminals in vivo [33,53,54] and are considered a relevant model system for studying human synaptic dysfunction in NDDs [55]. It is important to underline that the brain consumes about 25% of glucose available to the body, and in particular, synaptic activity requires a large amount of energy for its physiological needs [56,57]. This enormous energy demand depends on mitochondria, which are the primary generators of cell energy, as well as the principal site of ROS production [58], being also involved in the inflammatory processes [59]. In the central nervous system, the energy delivered by the mitochondria of the brain is greater than that of other organs and mitochondria have a crucial role as an essential energy source for cognitive functions. In particular, the mitochondria located in the synaptic region are closely involved in synaptic plasticity processes.

Therefore, we analysed the modulatory effect of butyrate on mitochondrial functions (detected by oxidative capacity and Seahorse analysis) in the cerebral cortex and in the synaptosomal fraction of the HFD mouse brain. Interestingly, treatment with butyrate was able to reverse the HFD-dependent alteration of the mitochondrial respiratory capacity selectively in the brain cortex of HFD-fed mice. Indeed, in the brain cortex of mice treated with butyrate compared with the HFD mice, state 3 respiration significantly increased in the presence of NADH-linked (pyruvate) and FADH-linked (succinate) as substrates. Also, for the same animal group, we observed a reduction in mitochondria efficiency indicated by a decreased degree of coupling. These results indicate that an increased amount of substrates needs to be burned to obtain ATP, as part of the proton gradient across the inner membrane is dissipated as heat [60,61]. The reduction in mitochondrial efficiency allows the mitochondrial membrane potential to remain below the critical threshold for ROS production [62,63]. According to this efficiency reduction, in the cerebral cortex of mice treated with butyrate we also observed an increased aconitase activity, an enzyme of the Krebs cycle sensitive to ROS, and a reduction in the levels of MDA, a lipid peroxidation index, and in ROS content.

Consistent with our previous results, HFD induced alterations in synaptic mitochondrial function. Indeed, in synaptosomes of HFD mice, by Seahorse analysis, we observed a reduction in basal respiration, maximal respiration and in ATP production. In addition, HFD induced an increased coupling efficiency associated with a reduction in proton leakage. These characteristics may be responsible for the increased oxidative stress and inflammatory status that we observed in the synaptic region of the HFD mice. Surprisingly, the administration of butyrate does not restore these respiratory parameters in the synaptosomal fraction, although it improves oxidative stress levels and inflammation parameters in this region. Therefore, these anti-inflammatory and antioxidant effects of butyrate in the synaptosomal fraction could be attributable to its well-known capacity of modulating histone deacetylase by an epigenetic mechanism [64], although further studies are necessary to deeply investigate this issue. Because it is well known that neuroinflammation influences the BDNF signalling in the brain [13], cerebral cortex expression of this neurotrophin was evaluated. In accordance with our previous results, we confirmed that HFD downregulated BDNF expression in this brain area. Interestingly, the administration of butyrate restores BDNF levels, in agreement with a recent study, demonstrating that butyrate upregulated BDNF expression and attenuated P-53, BAX and caspase cascades in the brain of HFD-fed mice [65]. Therefore, the observed protective effects of butyrate in this brain area are at least in part attributable to its ability to modulate BDNF expression.

## 5. Conclusions

In conclusion, the butyrate counteracts the inflammatory processes and oxidative stress induced by a HFD in the mouse brain cortex and synaptic area by promoting the inefficient use of mitochondrial energy substrates, generating heat instead of ATP, and by modulation of the BDNF pathway. Our findings suggest butyrate as a possible candidate in regulating neural plasticity and preventing the development of neurodegenerative disorders, emphasizing the involvement of mitochondria, crucial actors in cellular energy production.

## Data Availability

Data is contained within the article.
